# COVID-19 Alters Respiratory Function Associations in High-Level Athletes

**DOI:** 10.3390/medicina61091652

**Published:** 2025-09-11

**Authors:** Banu Kabak, Murat Erdoğan, Erkan Tortu, Gökhan Deliceoğlu, Celal Bulgay, Oktay Kızar, Giyasettin Baydaş, Attila Szabo

**Affiliations:** 1 Performance Laboratory, Department of Athlete Health Service Quality Standards, Ministry of Youth and Sports, 06820 Ankara, Türkiye; banu.kabak@gsb.gov.tr; 2 Exercise and Sport Science Department, Faculty of Health Sciences, Baskent University, 06790 Ankara, Türkiye; murate@baskent.edu.tr; 3 Department of Coaching Education, Faculty of Sport Sciences, Trabzon University, 61335 Trabzon, Türkiye; erkantortu@trabzon.edu.tr; 4 Department of Coaching Education, Faculty of Sport Sciences, Gazi University, 06560 Ankara, Türkiye; gokhandeliceoglu@gazi.edu.tr; 5 Faculty of Sports Science, Bingol University, 12000 Bingöl, Türkiye; cbulgay@bingol.edu.tr; 6 Faculty of Sports Science, Munzur University, 62000 Tunceli, Türkiye; oktaykizar@munzur.edu.tr; 7 Department of Physiology, Faculty of Medicine, Istanbul Medeniyet University, 34700 Istanbul, Türkiye; giyasettin.baydas@medeniyet.edu.tr; 8 Faculty of Health and Sport Sciences, Széchenyi István University, 9026 Győr, Hungary

**Keywords:** SARS-CoV-2, FVC, MIP, MEP, FEV_1_

## Abstract

*Background and Objectives*: Coronavirus disease 2019 (COVID-19) has affected multiple physiological systems, including respiratory function, which is critical for athletic performance. Although alterations in pulmonary dynamics have been observed in high-level athletes recovering from COVID-19, the effects on respiratory function remain unclear. In this context, the present study aimed to examine the impact of COVID-19 on the interrelationships among respiratory function parameters in high-level athletes. *Materials and Methods*: Sixty-eight high-level athletes participated in the present study, including 34 with a history of COVID-19 and 34 controls without prior infection. Respiratory function and respiratory muscle strength were assessed using a digital spirometer (Pony FX, Cosmed, Italy). Key variables included Forced Vital Capacity (FVC), Peak Expiratory Flow (PEF), Maximum Voluntary Ventilation (MVV), Forced Expiratory Volume in one second (FEV_1_), and Maximum Inspiratory/Expiratory Pressure (MIP/MEP). *Results*: High-level athletes with prior COVID-19 infection exhibited significant differences in the correlations (*p* < 0.05) between FVC and PEF, FVC and MVV, FEV_1_ and FEV_1_/FVC, and MIP and MVV compared to controls. *Conclusions*: These findings suggest that COVID-19 can disrupt the interrelationships among respiratory function parameters in high-level athletes, highlighting the need for further longitudinal investigations.

## 1. Introduction

Coronavirus disease (COVID-19), caused by severe acute respiratory syndrome coronavirus 2 (SARS-CoV-2), emerged in late 2019 as an unprecedented global health crisis with profound repercussions for the global community. In response to rapidly escalating infection rates, governments enforced stringent public health measures, including the postponement or cancellation of sporting events and training sessions [1]. These restrictions, compounded by the direct physiological effects of the virus, led to substantial declines in athletes’ physical conditioning and overall performance [2]. Beyond its acute clinical manifestations, severe COVID-19 has been associated with a “cytokine storm,” characterized by excessive pro-inflammatory cytokine release, systemic inflammation, multi-organ dysfunction, and increased mortality [3]. Elevated inflammatory cytokine levels have also been directly linked to diminished athletic performance, highlighting the relevance of this immune response for sport-specific outcomes [4].

Although athletes with mild to moderate infections are generally expected to recover fully, persistent symptoms such as coughing, exertional dyspnea, and fatigue are commonly reported during high-intensity training and competition. Moreover, COVID-19 may exacerbate pre-existing respiratory conditions frequently observed in athletes, including allergies, exercise-induced bronchoconstriction, and respiratory infections, thereby intensifying asthma-like manifestations and further impairing performance capacity [5,6]. These challenges underscore the importance of systematic post-COVID evaluations in athletes to ensure safe return-to-play (RTP) and to mitigate performance decline through evidence-based rehabilitation protocols [7].

Recent studies have broadened our understanding of COVID-19’s multifaceted impact on athlete health, particularly regarding cardiovascular, neuromuscular, and psychological sequelae. Zacher et al. (2023) identified alterations in autonomic regulation via heart rate variability metrics, reporting significantly higher resting heart rates and lower resting blood pressure in athletes with prior COVID-19 compared to controls, although no significant differences emerged during orthostatic stress [8]. Spyrou et al. (2021) documented impairments in sprint performance and select countermovement jump kinetics among futsal players following quarantine [9], while Fikenzer et al. (2021) demonstrated reductions in endurance capacity among handball players deprived of team training, despite adherence to individualized conditioning programs [10]. Complementing these findings, Kim et al. (2023) observed persistent fatigue, respiratory discomfort, and performance disturbances in over half of previously infected elite athletes, with female athletes and those experiencing widespread or cognitive symptoms being disproportionately affected [11]. Similarly, Beyer et al. (2023) reported reduced maximal performance and quality of life among post-COVID sedentary individuals, primarily driven by fatigue [12], while Karrer et al. (2022) highlighted the negative impact of COVID-19 restrictions on training load and mental health among Swiss elite athletes [13]. More recently, Cao et al. (2024) showed that while maximal and explosive power recovered within four weeks of infection, reactive power and initial force generation remained impaired, indicating delayed neuromuscular restoration [14]. Ribeiro et al. (2024) further emphasized the persistence of long-COVID symptoms such as dyspnea, palpitations, and exercise intolerance recommending individualized, multidisciplinary RTP frameworks [15].

Collectively, this growing body of literature confirms COVID-19’s detrimental influence on multiple physiological domains [16,17]. However, a critical gap persists, the respiratory dimension, particularly the interrelationship between key pulmonary parameters and performance outcomes, remains underexplored in high-level athletes [14,18]. This oversight is especially significant given that subtle impairments in respiratory function can substantially hinder high-intensity exertion and elevate health risks.

Accordingly, the present study aims to evaluate respiratory function and respiratory muscle strength in high-level athletes (shooting, archery, and bocce) with and without a history of COVID-19. By analyzing core pulmonary parameters and their interactions, this research seeks to provide novel insights into the chronic respiratory consequences of COVID-19 in athletic populations. The anticipated findings are expected to guide evidence-based rehabilitation strategies, enhance respiratory health, and refine safe and effective RTP protocols for elite athletes in the post-COVID era.

## 2. Materials and Methods

### 2.1. Ethical Approval

Ethical approval for the present study was obtained from the Gazi University Ethics Committee (Approval No. 2023/203). In addition, authorization to conduct the research was granted by the Education and Research Department of the relevant institution (Approval No. E-36592570-600-4502708). Written informed consent was obtained from all participants prior to data collection. The study was conducted in full compliance with the ethical standards of the institutional and national research committees, in accordance with the principles outlined in the Declaration of Helsinki 1975 (revised in 2013) and its subsequent amendments or equivalent ethical guidelines.

### 2.2. Sample Size Estimation

A priori power analysis was conducted using G*Power 3.1.9.7 to determine the minimum sample size required for adequate statistical power. The analysis was conducted with the Correlation: Point Biserial Model, corresponding to Pearson’s correlation analysis, which represented the primary statistical method for evaluating the relationships among respiratory parameters [19]. The parameters were set as follows: two-tailed test, effect size (r) = 0.343, α = 0.05, power (1-β) = 0.80, and pH0 = 0, resulting in a required minimum of 62 participants. To account for potential attrition, 68 athletes (34 with a history of COVID-19 and 34 without) were recruited, exceeding the calculated requirement. All participants completed the study, yielding an achieved statistical power of approximately 82%, sufficient to detect significant differences in respiratory function parameters between groups [20].

### 2.3. Participants

All athletes were recruited through the Ministry of Youth and Sports, Department of Athlete Health, Performance, and Service Quality Standards. Participation was entirely voluntary, and all athletes provided written informed consent after receiving detailed information about the study procedures. All assessments were conducted at least one month after the complete resolution of COVID-19–related symptoms.

The COVID-19 group consisted of 34 athletes (10 females, 24 males) who had a PCR-confirmed infection during the second half of 2021. This group included 23 shooters, 6 archers, and 5 bocce players. The control group comprised 34 athletes (7 females, 27 males) from the same disciplines, matched to the COVID-19 group in terms of sport type and sample size. The demographic characteristics of all participants are summarized in Table 1. None of the control athletes had a history of a positive PCR test, and all reported being free of respiratory or febrile illness for at least four weeks prior to data collection.

None of the athletes with COVID-19 required hospitalization. The most frequently reported symptoms included fever, headache, muscle and joint pain, and generalized body aches. Fourteen athletes experienced loss of taste and smell, whereas 20 did not report these symptoms. The mean time since infection was 5.2 ± 3.1 months; 26 athletes reported symptomatic infection, while 8 were asymptomatic. All participants had resumed full training for at least one month before testing.

Inclusion criteria for the COVID-19 group were a confirmed diagnosis via PCR testing, absence of any pre-existing respiratory condition prior to infection, and active participation in international competitions. Exclusion criteria included chronic respiratory disease, kidney or heart disease, neurological or psychiatric disorders, rheumatic or orthopedic conditions, musculoskeletal injury, and smoking. In addition, athletes who failed to resume regular training for at least one month after recovery or who did not provide informed consent were excluded from the study. Furthermore, it is noteworthy that all three disciplines included in the study (shooting, archery, and bocce) are precision-based sports that place particular emphasis on respiratory control and stability rather than high aerobic output, which aligns with the study’s objectives. Before data collection, all athletes were thoroughly informed about the study’s objectives, procedures, potential risks, and anticipated benefits. Voluntary participation was ensured for all individuals.

### 2.4. Data Collection Tools

#### 2.4.1. Personal Information Forms

Demographic characteristics of the participating athletes were obtained through a structured questionnaire encompassing items on gender, age, height, weight, sporting discipline, sport experience, year of COVID-19 diagnosis, and the interval before resuming sport participation. All data were collected in 2021 to ensure temporal consistency and accuracy.

#### 2.4.2. Respiratory Function Testing

Respiratory Function Testing (RFT) was performed using standardized procedures to ensure accuracy and reliability. Respiratory function test measurements were performed by a specialist physiotherapist who was trained on this subject and had practiced it many times. Athletes were seated in an upright position and fitted with a spirometer mouthpiece and nose clip to prevent air leakage, ensuring a tight lip seal [21]. Standardized respiratory maneuvers were executed through the mouthpiece [22], following preliminary trials to familiarize participants with the device. Each test was repeated three times with ≤3-min rest intervals, and the highest value was used for analysis [21]. Maximal Voluntary Ventilation (MVV) was assessed by instructing athletes to inhale and exhale forcefully, rapidly, and deeply for 12 s [23]. To minimize the risk of respiratory alkalosis, participants briefly held their breath after completing the MVV maneuver before the value was recorded.

#### 2.4.3. Respiratory Muscle Strength Testing

Respiratory muscle strength testing (RMST) was assessed using Maximum Inspiratory Pressure (MIP) and Maximum Expiratory Pressure (MEP) tests. For MIP, athletes exhaled fully to residual volume before performing a rapid, forceful inhalation [24]. For MEP, athletes inhaled to total lung capacity and then executed a rapid, forceful exhalation. Each test was performed three times with rest intervals between trials, and the highest value was retained for analysis.

#### 2.4.4. Statistical Analysis

Data normality was assessed using the Shapiro–Wilk test. For variables meeting the normality assumption, Pearson’s correlation coefficient was calculated to determine relationships between RFT parameters, including Forced Vital Capacity (FVC), Peak Expiratory Flow (PEF), and Maximal Voluntary Ventilation (MVV).

To account for potential confounding effects, gender, height, body weight, age, and sports experience were included as control variables, under the assumption that these factors exerted similar influences on both independent and dependent variables. To statistically compare the magnitude of correlation coefficients between the COVID-19 and control groups, Fisher’s z-test for independent correlations was employed. Additionally, 95% confidence intervals (CIs) for each correlation coefficient were calculated. All statistical analyses were performed using SPSS software (version 22.0; IBM Corp., Armonk, NY, USA), with statistical significance set at *p* < 0.05.

## 3. Results

Correlation analyses of respiratory function parameters in high-level athletes, stratified by COVID-19 history, are presented in Table 2, Table 3, Table 4 and Table 5.

In Table 2, athletes with a COVID-19 history showed a strong positive correlation between FVC (4.38 ± 1.06) and FEV_1_ (3.52 ± 0.75; *r* = 0.893), comparable to those without a history (FVC: 4.67 ± 1.02; FEV_1_: 3.81 ± 0.89; *r* = 0.881). FVC was weakly and negatively correlated with PEF (5.94 ± 1.57; *r* = −0.156) in the COVID-19 group, but moderately and positively correlated in the non-COVID group (6.35 ± 2.16; *r* = 0.658). Correlations between FVC and MVV were negligible and negative (124.44 ± 33.28; *r* = −0.048) in the COVID-19 group, versus moderate and positive (141.17 ± 37.80; *r* = 0.590) in the non-COVID group. No other notable parameter differences were detected.

In Table 3, FEV_1_/FVC ratios were slightly negative in the COVID-19 group (81.44 ± 6.87; *r* = −0.042) and moderately positive in the non-COVID group (81.37 ± 7.91; *r* = 0.634). FEV_1_–PEF correlations were negligible and negative (*r* = −0.078) in the COVID-19 group but strongly positive (*r* = 0.828) in the non-COVID group.

In Table 4, MIP–MVV correlations were slightly positive in the COVID-19 group (96.82 ± 27.71; *r* = 0.170) and moderately positive in the non-COVID group (100.32 ± 34.71; *r* = 0.510).

In Table 5, MEP–FVC correlations were low and positive in the COVID-19 group (127.02 ± 39.60; *r* = 0.100) and moderate and positive in the non-COVID group (113.38 ± 41.60; *r* = 0.407). MEP–FEV_1_ correlations were slightly positive (*r* = 0.195) in the COVID-19 group and moderately positive (*r* = 0.317) in the non-COVID group.

In Table 6, the direct statistical comparison of correlation coefficients between groups using Fisher’s z-test revealed significant alterations in the relationship between key respiratory parameters among athletes with a history of COVID-19. As presented in Table 6, the correlations between FVC and PEF (z = 3.82, *p* < 0.001), FVC and MVV (z = 3.02, *p* = 0.003), and FEV_1_ and PEF (z = 5.41, *p* < 0.001) were significantly weaker in the COVID-19 group compared to the control group. In contrast, the difference in the correlation between MIP and MVV across groups was not statistically significant (z = 1.56, *p* = 0.119). The 95% confidence intervals for the correlations in the COVID-19 group widely encompassed zero for these pairs, indicating a lack of a stable linear relationship, whereas the intervals for the control group were consistently above zero, confirming positive associations. These results provide statistical evidence that a prior COVID-19 infection disrupts the typical covariation of respiratory function measures in athletes.

## 4. Discussion

The present study demonstrates that COVID-19 markedly alters the interrelationships among respiratory function parameters in high-level athletes. Differences were observed in the strength of correlations between FVC and other indices, including PEF, MVV, and FEV_1_, depending on athletes’ COVID-19 history. Altered associations in FVC–PEF, FVC–MVV, FEV_1_–FEV_1_/FVC, FEV_1_–PEF, MIP–MVV, and MEP–FEV_1_ indicate that COVID-19 may impair both respiratory mechanics and muscle performance. Statistical analysis using Fisher’s z-test indicated that the strength of correlations between FVC–PEF, FVC–MVV, and FEV_1_–PEF was significantly reduced in athletes with a history of COVID-19, reflecting weaker interrelationships among these parameters.

These findings are consistent with previous research. Sarto et al. (2020) reported abnormal pulmonary function in COVID-19 patients at hospital discharge, suggesting potential long-term sequelae [1]. Similarly, Çelik et al. (2022) documented reductions in respiratory muscle strength and altered mechanics in volleyball players post-infection [25]. The persistence of a strong positive correlation between FVC and FEV_1_ in all athletes suggests preserved lung volumes and capacities, whereas the attenuation of other associations in athletes with a history of COVID-19 points to more subtle functional impairments.

The weakened correlation between PEF and FVC in athletes with prior infection suggests impaired expiratory muscle function and possible airway obstruction. This interpretation aligns with Sutherland et al. (2004), who reported that reductions in FEV_1_ and FVC may reflect both restrictive and obstructive conditions [26]. Rajpal et al. (2021) also described cardiovascular and pulmonary complications in athletes following COVID-19, emphasizing the multifaceted impact of the disease [5]. Furthermore, the observed changes in correlations involving MIP and MEP may reflect compensatory mechanisms in some athletes, while others show reduced respiratory muscle strength, as noted by Çelik et al. (2021) [25]. Such variability underscores the importance of individualized rehabilitation protocols.

FEF25–75% is a sensitive marker of peripheral airway obstruction, and reductions in this parameter, even when FEV_1_ and FVC are preserved, may indicate early-stage obstructive pathology [6,27]. A particularly notable finding of our study was that the correlation between FEF25–75% and FVC was evident only in athletes without a history of COVID-19. In contrast, this relationship was absent in athletes with prior infection, suggesting that small airway function may be especially vulnerable to residual impairment.

Similarly, the diminished correlations between FEV_1_ and PEF, and between FEV_1_ and MEP, point to reductions in expiratory flow and muscle strength, both of which are critical for maintaining respiratory efficiency. FEV_1_, influenced by lung volumes, elastic recoil, respiratory muscle strength, and effort, is a key predictor of obstruction severity [4]. The reduced strength of these correlations in athletes with COVID-19 history highlights potential disruptions in these physiological determinants. When examining the relationship between FVC and MIP, a significant correlation was found in athletes without prior infection but was absent in those with COVID-19 history. This difference may be explained by higher MIP values among unaffected athletes. Moreover, MVV, a diagnostic indicator of respiratory muscle weakness [23], demonstrated strong correlations with MIP and MEP in athletes without infection, whereas these relationships were weakened in athletes with prior COVID-19. These findings emphasize the need for further research on long-term respiratory outcomes in athletes, including diffusion capacity and static lung volumes, to provide a more comprehensive understanding of COVID-19’s effects.

The present study is notable in that it examined athletes with PCR-confirmed COVID-19 and explored the interrelationships among respiratory parameters, offering novel insights into the functional consequences of infection in this population.

Nevertheless, several limitations should be acknowledged. The inclusion of a limited range of sports disciplines may affect the generalizability of findings. Female athletes were underrepresented, and sex-specific analyses could not be performed. Training load data were not collected, and information on disease severity (e.g., hospitalization, oxygen saturation) was lacking. Additionally, the COVID-negative status of control participants was based on self-report rather than PCR testing, which may have introduced misclassification. Furthermore, information on the exact timing of infection, vaccination status, and circulating variants was not collected in this study. This represents a key limitation, as these factors are known to influence pulmonary outcomes, and should therefore be taken into account when interpreting the findings. Finally, respiratory function outcomes were not directly linked to performance metrics, limiting the interpretation of practical implications for athletic performance.

Future research should address these limitations by incorporating larger and more diverse athlete samples, ensuring balanced sex representation, and collecting detailed clinical and training load data. Longitudinal studies including diffusion capacity, static lung volumes, and sport-specific performance measures will be essential to clarify the long-term impact of COVID-19 on athletes’ respiratory health.

## 5. Conclusions

The present study provides evidence that COVID-19 modifies the correlations among respiratory function parameters in elite athletes. While certain lung volumes such as FVC and FEV_1_ remain preserved, the weakening of associations involving PEF, MVV, and respiratory muscle indices reveals subtle but meaningful disruptions in respiratory function.

These findings highlight the importance of comprehensive respiratory assessments in post-COVID athletes and point to the need for targeted rehabilitation strategies to support recovery. Considering the unique physiological demands placed on elite athletes, further research is needed to determine the long-term consequences of COVID-19. Large-scale, longitudinal studies and clinical trials will be critical to guide effective monitoring and intervention strategies that enable athletes to restore optimal respiratory function and sustain peak performance following infection.

## Figures and Tables

**Table 1:** Demographic information of the research group.

COVID-19 History	Yes (*n* = 34) M ± SD	No (*n* = 34) M ± SD	Total (*n* = 68) M ± SD
Age (year)	18.00 ± 3.17	18.02 ± 3.45	18.01 ± 3.28
Height (cm)	171.61 ± 8.63	175.85 ± 8.30	173.73 ± 8.67
Weight (kg)	68.94 ± 13.25	71.73 ± 11.21	70.33 ± 12.26
Sports Experience (year)	4.79 ± 2.44	5.05 ± 2.33	4.95 ± 2.37
Training Duration (week/day)	4.47 ± 0.13	4.47 ± 0.13	4.47 ± 0.94
Training Duration (days/hours)	3.79 ± 0.12	3.79 ± 0.12	3.79 ± 0.87

M: Mean; SD: Std. Deviation.

**Table 2:** Correlation analysis results between FVC and other parameters of high-level athletes with and without COVID-19 history.

**With COVID-19 History**		**FVC**	**FEV_1_**	**FEV_1_/FVC**	**PEF**	**FEF_25–75_**	**MVV**	**MIP**	**MEP**
FEV_1_	r	1.000	0.893	−0.460	−0.156	0.086	−0.048	0.133	0.100
	*p*		0.001	0.006	0.210	0.328	0.402	0.245	0.302
**Without COVID-19 History**		**FVC**	**FEV_1_**	**FEV** **_1_/FVC**	**PEF**	**FEF_25–75_**	**MVV**	**MIP**	**MEP**
FEV_1_	r	1.000	0.881	0.202	0.658	0.568	0.590	0.375	0.407
	*p*		0.001	0.147	0.001	0.001	0.001	0.023	0.014

FVC: Forced Vital Capacity, FEV_1_: Forced Expiratory Volume in One Second, FEV_1_/FVC: Ratio of Forced Expiratory Volume in One Second to Forced Vital Capacity, PEF: Peak Expiratory Flow, FEF: Forced Expiratory Flow, MVV: Maximum Voluntary Ventilation, MIP: Maximum Inspiratory Pressure, MEP: Maximum Expiratory Pressure.

**Table 3:** Correlation analysis results between FEV_1_ and other parameters of high-level athletes with and without COVID-19 history.

**With COVID-19 History**		**FVC**	**FEV_1_**	**FEV_1_/FVC**	**PEF**	**FEF_25–75_**	**MVV**	**MIP**	**MEP**
FEV_1_	r	0.893	1.000	−0.042	0.078	0.461	0.031	0.163	0.195
	*p*	0.001		0.414	0.343	0.006	0.437	0.199	0.156
**Without COVID-19 History**		**FVC**	**FEV** **_1_**	**FEV_1_/FVC**	**PEF**	**FEF_25–75_**	**MVV**	**MIP**	**MEP**
FEV_1_	r	0.881	1.000	0.634	0.828	0.840	0.585	0.379	0.317
	*p*	0.001		0.001	0.001	0.001	0.001	0.021	0.047

FVC: Forced Vital Capacity, FEV_1_: Forced Expiratory Volume in One Second, FEV_1_/FVC: Ratio of Forced Expiratory Volume in One Second to Forced Vital Capacity, PEF: Peak Expiratory Flow, FEF: Forced Expiratory Flow, MVV: Maximum Voluntary Ventilation, MIP: Maximum Inspiratory Pressure, MEP: Maximum Expiratory Pressure.

**Table 4:** Correlation analysis results between MIP and other parameters of high-level athletes with and without COVID-19 history.

**With COVID-19 History**		**FVC**	**FEV_1_**	**FEV_1_/FVC**	**PEF**	**FEF_25–75_**	**MVV**	**MIP**	**MEP**
MIP	r	0.133	0.163	−0.040	0.236	0.111	0.170	1.000	0.670
	*p*	0.245	0.199	0.419	0.109	0.283	0.189		0.001
**Without COVID-19 History**		**FVC**	**FEV_1_**	**FEV_1_/FVC**	**PEF**	**FEF_25–75_**	**MVV**	**MIP**	**MEP**
MIP	r	0.375	0.379	0.252	0.192	0.291	0.510	1.000	0.731
	*p*	0.023	0.021	0.094	0.160	0.063	0.002		0.001

FVC: Forced Vital Capacity, FEV_1_: Forced Expiratory Volume in One Second, FEV_1_/FVC: Ratio of Forced Expiratory Volume in One Second to Forced Vital Capacity, PEF: Peak Expiratory Flow, FEF: Forced Expiratory Flow, MVV: Maximum Voluntary Ventilation, MIP: Maximum Inspiratory Pressure, MEP: Maximum Expiratory Pressure.

**Table 5:** Correlation analysis results between MEP and other parameters of high-level athletes with and without COVID-19 history.

**With COVID-19 History**	**FVC**	**FEV_1_**	**FEV_1_/FVC**	**PEF**	**FEF_25–75_**	**MVV**	**MIP**	**MEP**
MEP	r	0.100	0.195	0.103	0.549	0.087	0.269	0.670
	*p*	0.302	0.156	0.298	0.001	0.328	0.079	0.001
**Without COVID-19 History**	**FVC**	**FEV_1_**	**FEV_1_/FVC**	**PEF**	**FEF_25–75_**	**MVV**	**MIP**	**MEP**
MEP	r	0.407	0.317	0.100	0.049	0.269	0.470	0.731
	*p*	0.014	0.047	0.303	0.401	0.079	0.005	0.001

FVC: Forced Vital Capacity, FEV_1_: Forced Expiratory Volume in One Second, FEV_1_/FVC: Ratio of Forced Expiratory Volume in One Second to Forced Vital Capacity, PEF: Peak Expiratory Flow, FEF: Forced Expiratory Flow, MVV: Maximum Voluntary Ventilation, MIP: Maximum Inspiratory Pressure, MEP: Maximum Expiratory Pressure.

**Table 6:** Statistical comparison of selected correlation coefficients between respiratory parameters in high-level athletes with and without a history of COVID-19 using Fisher’s z-test.

Correlation Pair	With COVID-19 (*n* = 34)	Without COVID-19 (*n* = 34)	Fisher’s z-Test	
	r [95% CI]	r [95% CI]	z	*p*
FVC-PEF	−0.156 [−0.456, 0.172]	0.658 [0.408, 0.816]	3.82	0.001
FVC-MVV	−0.048 [−0.368, 0.282]	0.590 [0.318, 0.771]	3.02	0.003
FEV_1_-PEF	−0.078 [−0.392, 0.252]	0.828 [0.681, 0.910]	5.41	0.001
MIP-MVV	0.170 [−0.162, 0.471]	0.510 [0.202, 0.728]	1.56	0.119

CI: confidence interval; FVC: Forced Vital Capacity; PEF: Peak Expiratory Flow; MVV: Maximum Voluntary Ventilation; FEV_1_: Forced Expiratory Volume in 1 s; MIP: Maximum Inspiratory Pressure.

## Data Availability

The data supporting this study’s findings are not publicly available due to privacy reasons but are available from the corresponding author.
